# Mitigating Feelings of Loneliness and Depression by Means of Web-Based or Print-Based Physical Activity Interventions: Pooled Analysis of 2 Community-Based Intervention Trials

**DOI:** 10.2196/36515

**Published:** 2022-08-09

**Authors:** Sonia Lippke, Tiara Ratz, Franziska Maria Keller, Dennis Juljugin, Manuela Peters, Claudia Pischke, Claudia Voelcker-Rehage

**Affiliations:** 1 Psychology & Methods Jacobs University Bremen Bremen Germany; 2 Department of Reproductive Endocrinology University Hospital Zurich (USZ) University of Zurich (UZH) Zurich Switzerland; 3 Psychology Universitaet Bremen Bremen Germany; 4 Leibniz Institute for Prevention Research and Epidemiology–BIPS Bremen Germany; 5 Institute of Medical Sociology, Centre for Health and Society, Medical Faculty Heinrich Heine University Duesseldorf Duesseldorf Germany; 6 Department of Neuromotor Behavior and Exercise, Institute of Sport and Exercise Sciences University of Muenster Muenster Germany

**Keywords:** physical activity, older adults, intervention, loneliness, depression, eHealth, mobile health, mHealth

## Abstract

**Background:**

Physical activity (PA) is associated with benefits, such as fewer depressive symptoms and loneliness. Web- and print-based PA interventions can help older individuals accordingly.

**Objective:**

We aimed to test the following research questions: Do PA interventions delivered in a web- or print-based mode improve self-reported PA stage of change, social-cognitive determinants of PA, loneliness, and symptoms of depression? Is subjective age a mediator and stage of change a moderator of this effect?

**Methods:**

Overall, 831 adults aged ≥60 years were recruited and either allocated to a print-based or web-based intervention group or assigned to a wait-list control group (WLCG) in 2 community-based PA intervention trials over 10 weeks. Missing value imputation using an expectation-maximization algorithm was applied. Frequency analyses, multivariate analyses of variance, and moderated mediation analyses were conducted.

**Results:**

The web-based intervention outperformed (47/59, 80% of initially inactive individuals being adopters, and 396/411, 96.4% of initially active individuals being maintainers of the recommended PA behavior) the print-based intervention (20/25, 80% of adopters, and 63/69, 91% of maintainers) and the WLCG (5/7, 71% of adopters; 141/150, 94% of maintainers). The pattern regarding adopters was statistically significant (web vs print *Z*=–1.94; *P*=.02; WLCG vs web *Z*=3.8367; *P*=.01). The pattern was replicated with stages (*χ*^2^_4_=79.1; *P*<.001; contingency coefficient 0.314; *P*<.001); in the WLCG, 40.1% (63/157) of the study participants moved to or remained in action stage. This number was higher in the groups receiving web-based (357/470, 76%) or print-based interventions (64/94, 68.1%). A significant difference was observed favoring the 2 intervention groups over and above the WLCG (*F*_19, 701_=4.778; *P*<.001; *η*^2^=0.098) and a significant interaction of time and group (*F*_19, 701_=2.778; *P*<.001; *η*^2^=0.070) for predictors of behavior. The effects of the interventions on subjective age, loneliness, and depression revealed that both between-group effects (*F*_3, 717_=8.668; *P*<.001; *η*^2^=0.018) and the interaction between group and time were significant (*F*_3, 717_=6.101; *P*<.001; *η*^2^=0.025). In a moderated mediation model, both interventions had a significant direct effect on depression in comparison with the WLCG (web-based: *c*′ path −0.86, 95% CI −1.58 to −0.13, SE 0.38; print-based: *c*′ path −1.96, 95% CI −2.99 to −0.92, SE 0.53). Furthermore, subjective age was positively related to depression (*b* path 0.14, 95% CI 0.05-0.23; SE 0.05). An indirect effect of the intervention on depression via subjective age was only present for participants who were in actor stage and received the web-based intervention (*ab* path −0.14, 95% CI −0.34 to −0.01; SE 0.09).

**Conclusions:**

Web-based interventions appear to be as effective as print-based interventions. Both modes might help older individuals remain or become active and experience fewer depression symptoms, especially if they feel younger.

**Trial Registration:**

German Registry of Clinical Trials DRKS00010052 (PROMOTE 1); https://tinyurl.com/nnzarpsu and DRKS00016073 (PROMOTE 2); https://tinyurl.com/4fhcvkwy

**International Registered Report Identifier (IRRID):**

RR2-10.2196/15168

## Introduction

### Web-Based and Printed Intervention Material

Loneliness is a key element, along with lifestyle factors such as physical activity (PA), which is interrelated with health and well-being [1-3]. Although the concept of loneliness has a long history, many concerns exist that modern times increase social isolation among older people [3,4]. Since the beginning of the COVID-19 (SARS-CoV-2) pandemic, social isolation and loneliness have received heightened attention [1-4]. Reasons for elevated concerns related to loneliness during the COVID-19 pandemic were seen because of the required social distancing (ie, because of distancing rules, citizens were not allowed to be in close physical contact with others or generally to come together). In addition, people limited their personal contacts and followed stay-at-home orders and face mask mandates in public [2-4]. Steps are needed to bridge the gap between the necessary actions for public health, individual health, and well-being. Such bridging can be done by means of web-based and print-based interventions in comparison with no support (ie, a wait-list control group [WLCG]).

Dickens et al [5] performed a systematic review of interventions targeting social isolation among older adults. They found that 86% of the interventions aimed at supporting activities (social activities and PAs) were effective [5]. Specifically, these activities were comprised of group and psychosocial accomplishments and included besides exercise and PA also arts, discussion rounds, therapeutic writing, group therapy, reading to children, lectures, assistance with organizing social behavior, outings, mutual help networks as well as leisure and cultural events with different durations [5]. Activities, especially social components and PA, are key to preventing or overcoming social isolation and loneliness [4,6]. However, only 25% of internet training interventions have revealed a successful reduction in social isolation among older adults [5].

In conclusion, the evidence demonstrates that interventions fostering physical exercise and PA can improve mood; increase physical, social, and cognitive activities; and decrease social isolation [7]. However, little is known about the delivery mode of the intervention (eg, the comparison of internet-based training interventions and traditional print-based interventions), as well as the mechanisms that may explain possible differential effects. Therefore, this study addressed this open question. The research question was whether more adults reported changing their PA behavior if they received the web-based or print-based intervention than those who received the control condition.

A previous study by Boekhout et al [8] revealed the benefits of a printed delivery method compared with a web-based version. Specifically, the authors found higher participation and lower attrition rates in this group [8]. Golsteijn et al [9] compared printed materials with web-based materials in terms of cost-effectiveness and cost-utility to promote PA among adults aged ≥50 years. The results revealed that the print-based material was most cost-effective in terms of increasing PA and could also contribute to better overall health at the population level [9]. However, little is known about the effectiveness in terms of loneliness and social-cognitive predictors, what actually explains the effects of the intervention, and in whom and how the intervention works [10]. Depression can be an important component of mental health [1], whereas a central factor of well-being and successful aging is feeling subjectively fit [7]. Thus, this study investigated whether a web-based or print-based PA intervention improved outcomes such as social-cognitive predictors of PA behavior change, subjective age, feelings of loneliness, and symptoms of depression in comparison with a control group. Furthermore, we examined whether mediating and moderating mechanisms exist. Conceivable mechanisms will be outlined in the following sections to set the stage for this study.

### Potential of PA Interventions

PA is imperative for health and well-being at any age and is increasing in importance with older age [11,12]. Approximately half of the population will be aged >60 years by 2030 [13]. Consequently, it is important to improve the health of this population. Regular PA, particularly cardiovascular training (also called endurance training), is considered to have enormous potential for maintaining and improving the health and well-being of older adults [14,15]. Following the World Health Organization (WHO) recommendations, PA should be conducted for ≥150 minutes per week with moderate to vigorous PA (MVPA) in bouts of at least 10 minutes to improve and maintain health [13].

Cross-sectionally, more PA is related to better health and vice versa, and the improvement of a healthy lifestyle has been demonstrated to pay off in terms of increasing or recovering health [16,17]. Olson and McAuley [18] demonstrated the effectiveness of an intervention, including walking exercise (endurance training) and theory-based group workshops, aimed at improving the PA level of older adults in the short and long term.

An important limitation of PA programs for older adults is that they are often unattractive to older adults [19]. This might be overcome by addressing the individual-level characteristics and (technology-based) preferences of participants [20]. Concerning the overall uptake of PA and sports offerings, demographic and socioeconomic individual-level differences have been shown to be relevant in past studies [21-23]. Those who are already actively involved in PA and sports are more likely to participate [23-25]. However, some people may also experience that being active operates as a barrier to adopting activities such as a new physical exercise program [26], showing that previous behavior is yet another predictor that depends on individual circumstances. A study of older adults’ specific requirements for PA class meetings also revealed sex-specific differences. For example, men, in contrast to women, were more critical of group activities [27]. Further identification of how and why interventions work can help the development and organization of attractive future health interventions [19,20]. A theoretical framework that might explain the differences based on baseline characteristics such as previous experience is described in the following section.

### Theory-Based Interventions and Social-Cognitive Predictors of PA Behavior Change

Research comparing the effectiveness of theory-based and non–theory-based health behavior change interventions has demonstrated a higher potential for theory-based approaches to effectively promote PA [28-30], although not consistently [31]. However, overall, it should be noted that health behavior change interventions to improve PA are very heterogeneous with regard to theoretical approaches, designs, and effectiveness. In addition, some interventions have only been found to produce small to moderate effects [32-34]. For example, an aggregated effect of Cohen *d*=0.27 was determined by Rhodes et al [34] in a high-level overview of published reviews of the literature, which has been interpreted as small but meaningful. This shows that theoretical frameworks that take further relevant parameters and pathways into account are needed for the design of PA interventions.

Social-cognitive variables are imperative for predicting active behavior change [35]. Knowledge of such variables enables the design of interventions. For example, key social-cognitive variables are described in the Health Action Process Approach (HAPA) [35]. The HAPA is a theory that organizes different social-cognitive variables into a meaningful structure [35]. The HAPA has two layers: a continuum layer with social-cognitive variables and a stage layer with the stages of change. The HAPA assumes three different stages of change: the nonintenders stage with its processes that lead to a behavioral intention, the intenders stage with postintentional volition processes that lead to the actual health behavior, and the action stage where the goal behavior is performed.

Within different stages, different patterns of social-cognitive predictors may emerge. In the nonintenders stage, a person develops the intention to act. In this phase, risk perception is seen as a distal antecedent (eg, “I am at risk for cardiovascular disease”). Risk perception in itself is insufficient to enable a person to form an intention. Rather, it sets the stage for a contemplation process and further elaboration of thoughts on consequences and competencies. Similarly, positive outcome expectancies (eg, “If I exercise five times per week, I will reduce my cardiovascular risk”) are chiefly seen as important in the motivation phase when a person balances the pros and cons of certain behavioral outcomes. Furthermore, one needs to believe in one’s capability to perform the desired action (perceived self-efficacy; eg, “I am capable of adhering to my exercise schedule despite the temptation to watch TV”). Perceived self-efficacy operates in concert with positive outcome expectancies, both of which contribute substantially to the formation of intention. Both beliefs are needed to form intentions to adopt difficult behaviors, such as regular physical exercise.

After forming an intention, the volitional phase is entered. Once a person is inclined to adopt a particular health behavior, the *good intention* must be transformed into detailed instructions on how to perform the desired action. As soon as an action is initiated, it must be maintained. This is not achieved through a single act of will but involves self-regulatory skills and strategies. Thus, the postintentional phase should be further broken down into more proximal factors, such as planning, action control, social support, and recovery self-efficacy.

Social support is a factor that reflects the barriers and resources part of the HAPA model: support represents a resource, and the lack of it can be a barrier to adopting or maintaining health behaviors. Instrumental, emotional, and informational social support can enable the adoption and continuation of behaviors [35]. The theoretical assumptions not only improve the prediction of behavior but also allow for designing of interventions more effectively by tailoring the intervention components to the needs of the recipient and, finally, enhance participation. The relevant factors are described in the following sections.

### Tailored Web-Based Intervention

Tailoring is a key aspect of making interventions more effective, not only by considering the users’ stage of change but also by matching the users’ needs. For instance, such needs can be that participants prefer self-monitoring and activity tracking as components of their intervention (eg, by digital formats, as found by Powell et al [36]).

Digital modes have much more potential than paper-based intervention modes as they provide more options for personalization. At the same time, information can be delivered in different forms, including textual, visual, and audiovisual information to suit individual preferences and abilities [37]. However, when preferences are considered, older people in particular like print formats better [38] and accordingly might benefit more from it. However, this requires more systematic research.

The tailoring of interventions is a method that aims to meet the needs of all individuals more appropriately. However, meeting all these different needs is typically challenging. Therefore, it is necessary to evaluate whether all individuals benefit equally. For instance, in a previous study [39], it was found that participants who were not sufficiently physically active before the study (nonintenders and intenders in comparison with actors) found the intervention useful. In another study [40], the printed method was more effective than the internet method in participants with a high baseline intention for PA (intenders). Thus, the question remains of whether the intervention is moderated by the stage of change in endurance activities. Other needs may be interrelated with sociodemographic characteristics such as age, which necessitates further elaboration, as will be described in the following sections.

### Aging, Loneliness, and Subjective Age

Aging is typically stereotyped as feeling lonely. However, loneliness is not related to older age but the opposite—younger cohorts feel more lonely than older cohorts [1]. The aging population is at higher risk of other health-related challenges [1,3,4]. Aging processes and the health of older adults are highly important. Many older adults experience more health limitations and an increased burden, such as falling upon their caregivers [41]. In addition, older adults might have the highest risk of inactive lifestyles because of their reduced functioning [42].

Aging theories posit that older adults prefer to exercise with other individuals instead of exercising alone [43]. Accordingly, blended web-based and print interventions for older individuals promoting PA proved to be effective [44-46] as web-based materials would typically be used more for unaccompanied modes. However, whether print and web-based materials are beneficial for older adults’ health (eg, symptoms of depression), well-being (or the opposite, eg, loneliness), health behavior, and its predictors requires further investigation.

Typically, calendrical and subjective ages are distinguished [47,48]. Calendrical age is determined by the date of birth [38]. In contrast, if a person is asked how old they feel, then the perceived or subjective age can be determined [47]. The latter is associated with health status and well-being, as well as with behavioral, cognitive, and biological processes, including frailty [47].

Although calendrical age cannot be changed, subjective age contains many options for interventions: individuals who feel younger are better off and more optimistic [48]. Previous studies have demonstrated that interventions can improve subjective age and general health status or even reverse frailty [47]. The question remains whether the effect of a PA intervention is mediated by subjective age; thus, this study investigated this in further detail.

### Interrelations and Stage of Change

Loneliness and mental health issues, such as depression, are interrelated [1]. Fortuna et al [10] summarized that older adults can benefit from digital services to overcome their mental health limitations (such as loneliness and depression). Moreover, PA can help reduce depression and loneliness using mastery experience and self-regulation with regard to physical perceptions and repairing interpersonal skills and relationships [6]. Accordingly, the PA intervention group (IG) allocation should reduce the likelihood of depressive symptoms and loneliness.

However, much is still unresearched, such as whether internet-based services are as good as, or better than, traditional services for older individuals’ mental health. Although the advantages are obvious, the effects on outcomes such as loneliness and symptoms of depression still need more systematic attention, which will be addressed in this study.

**Figure 1:**
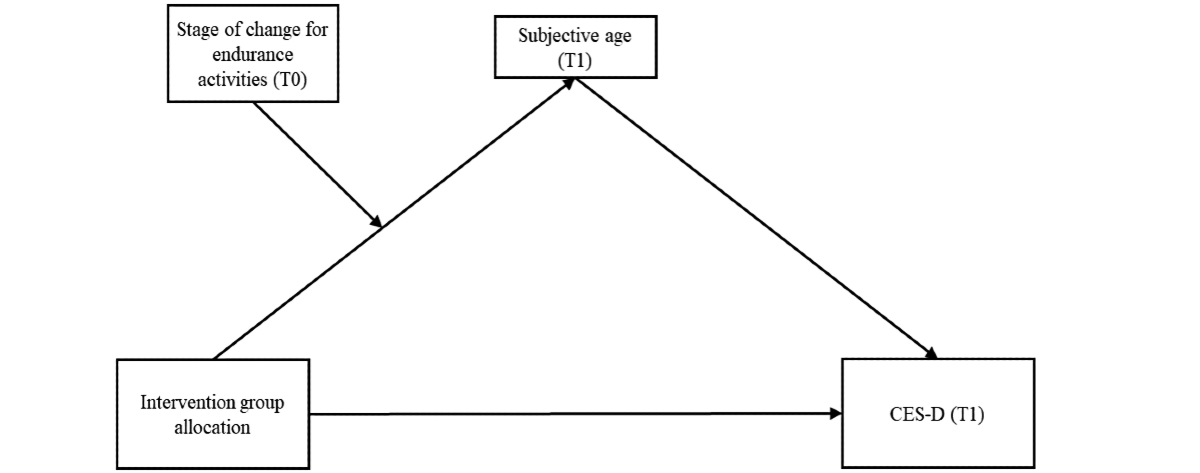
Proposed moderated mediation model for depression. CES-D: Center for Epidemiologic Studies Depression; T0: time point 0; T1: time point 1.

With respect to intervention studies, it is assumed that the assignment of participants to specific study arms with different forms of content would have an effect on symptoms of depression and that this effect is mediated by subjective age and moderated by the stage of change in endurance training (Figure 1).

**Figure 2:**
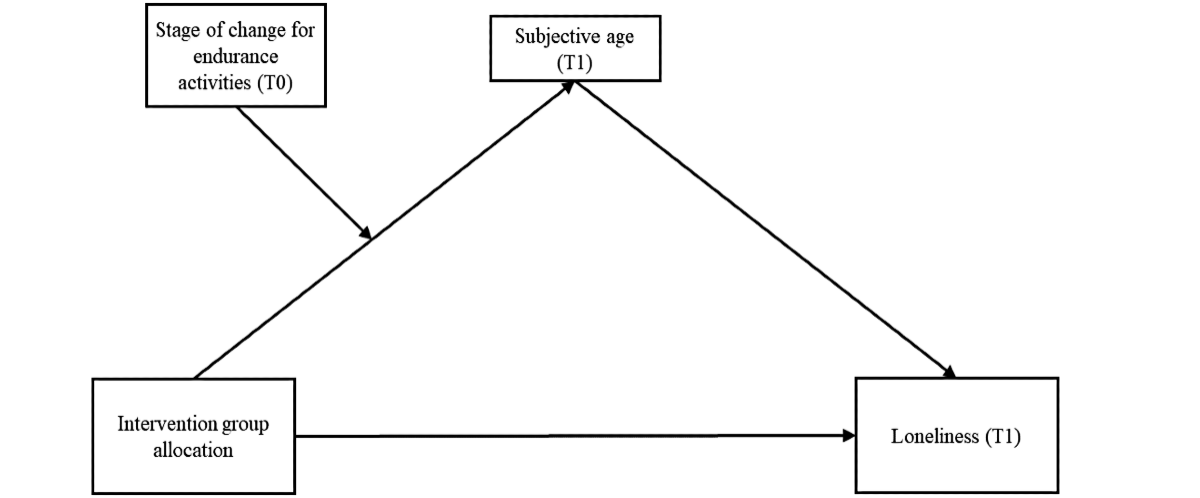
Proposed moderated mediation model for loneliness. T0: time point 0; T1: time point 1.

Furthermore, the question remains as to whether the same relationship with loneliness as a dependent variable would be feasible (Figure 2). Accordingly, research is required regarding the key question of whether the intervention effects on loneliness and symptoms of depression depend on subjective age and whether the stage of change for endurance activities affects this effect. Thus, the hypotheses described in the following sections were investigated.

### Goals of This Study

The goal of this study was to test the following research questions: do interventions delivered in a web or print mode improve self-reported PA stage of change, social-cognitive determinants of PA, feelings of loneliness, and symptoms of depression, and in this effect, does subjective age act as a mediator and stage of change act as a moderator?

The following hypotheses were tested:

1. The proportion of older adults who self-report a PA behavior change is higher in the web-based and print-based PA interventions than in the respective control conditions.
2. Compared with the control condition, the web-based and print-based PA interventions improve social-cognitive predictors of PA behavior change, subjective age, feelings of loneliness, and symptoms of depression.
3. The intervention’s effect on feelings of loneliness is mediated by subjective age, and this mediation is moderated by the stage of change for endurance activities (moderated mediation).
4. The intervention effect on symptoms of depression (Center for Epidemiologic Studies Depression [CES-D] score) is mediated by subjective age, and this mediation is moderated by the stage of change in endurance activities (moderated mediation).

## Methods

### Overview

The PROMOTE study comprised 2 cohorts: PROMOTE 1 and 2. In this study, web- and print-based programs to promote PA in community-dwelling older adults were developed, analyzed, and evaluated according to multiple theoretical models and intervention effects using randomized intervention trials [49-54]. These were conducted as part of the interdisciplinary Physical Activity and Health Equity: Primary Prevention for Healthy Aging research network [55].

The first trial (2015-2018, PROMOTE 1) compared the effects of 2 web-based interventions with a wait-listed control condition, whereas the second trial (2018-2021, PROMOTE 2) compared adapted versions of the web-based interventions (the program was adapted to initially inactive older adults) with a print-based PA intervention. For the analyses in this study, the groups receiving the web-based intervention in PROMOTE 1 and 2 were combined. The plan for pooling the data of the 2 intervention studies is described in the study protocol of PROMOTE 2 [51]. Accordingly, measures with the intervention design were taken to synchronize the different interventions from the beginning of PROMOTE 2: interventions did not significantly differ in their content and with the levels of recommended activity levels [51]. Measures were matched for PROMOTE 1 and 2 to pool data from both trial periods for the overarching analyses.

### Ethics Approval

PROMOTE 1 was approved by the Ethics Committee of the Technical University of Chemnitz, Faculty of Behavioral and Social Sciences, on July 14, 2015, with the ethics approval number: V-099-17-HS-CVR-PROMOTE-03072015. Ethics approval for PROMOTE 2 was obtained from the Medical Association in Bremen on July 3, 2018, with the ethics approval number 635. The trials were conducted in accordance with the ethical principles of the American Psychological Association and the 1964 Declaration of Helsinki and its later amendments of comparable ethical standards. All participants were fully informed about the study and provided informed consent.

### Recruitment

Detailed information regarding data collection (recruitment and randomization strategies) can be obtained from the studies by Muellmann et al [49] and Pischke et al [51]. Briefly, in 2016 (PROMOTE 1) and 2018 (PROMOTE 2), random samples of n=8299 older adults aged 65 to 75 years and n=3492 older adults aged ≥60 years and living independently (without assisted living) were selected by the residents’ registration offices from municipalities in the Bremen metropolitan region and invited to participate by mail. In addition, both study phases were promoted in the local press, as well as via prior discussions with the research staff with an offer to enroll voluntarily. Eligibility for study participation, which is described in detail in the published study protocols [49,51], was determined through computer-assisted telephone interviews with trained study nurses.

The main inclusion criteria were being aged 65 to 75 years in PROMOTE 1 and ≥60 years in PROMOTE 2, as well as living independently, having basic knowledge of German, being able to walk without a walking aid, participation in study assessments and weekly group meetings without external assistance, and providing informed consent.

The exclusion criteria were as follows: a medical condition or diagnosis prohibiting PA, severe visual or other impairments, implanted cardiac devices, or occasional syncopal episodes leading to exclusion or cognitive impairment (Mini-Mental State Examination [MMSE] <25 in PROMOTE 1 and MMSE second edition: MMSE-2: Brief Version [MMSE-2:BV] score <13 in PROMOTE 2). Individuals were excluded from the study if they were planning a vacation during the intervention period, had certain medical conditions or severe health impairments, or did not have a mobile device or internet access. As the results of the first study indicated that predominantly already active individuals participated in the study, the exclusion criteria for PROMOTE 2 were modified, and individuals were excluded if they reported being regularly physically active for at least 2.5 hours per week for >1 year before the start of the study. Potential study participants for PROMOTE 2 were excluded if they had already participated in PROMOTE 1 [52].

Finally, participants were randomly assigned to the following study arms:

1. In PROMOTE 1 (N=589), to either a web-based intervention with subjective PA self-monitoring (211/589, 35.8%), web-based intervention with subjective and objective PA self-monitoring (198/589, 33.6%), or WLCG (180/589, 30.6%) [49]
2. In PROMOTE 2 (N=242), to a print-based intervention with subjective PA self-monitoring via a printed PA pyramid (113/242, 46.7%) and web-based intervention with subjective PA self-monitoring via a web-based PA pyramid (129/242, 53.3%); approximately 29.5% (38/129) of the latter were randomly selected and received a PA tracker (objective PA self-monitoring) in addition [51]

**Figure 3:**
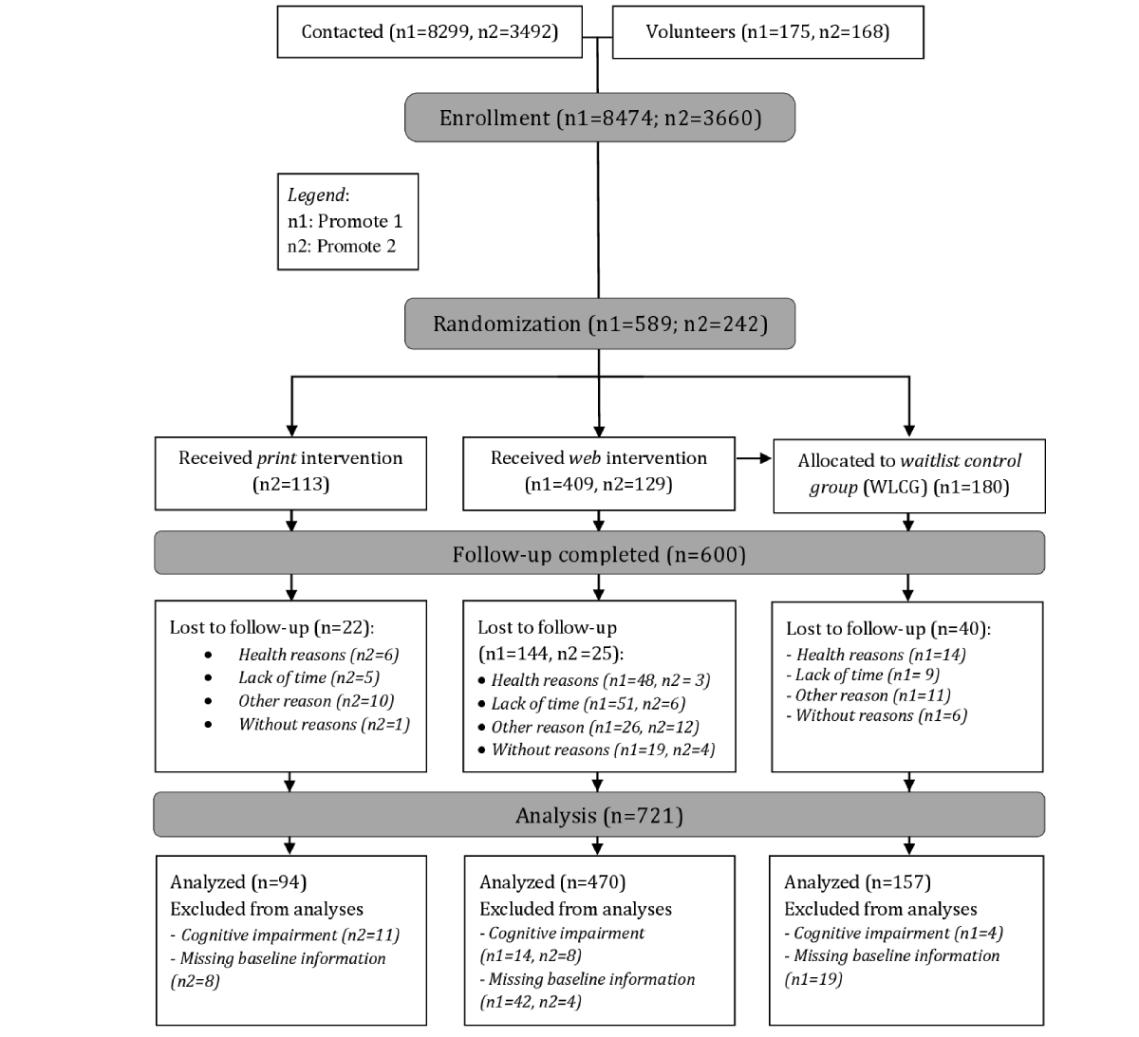
Study flow chart.

In total, 831 individuals were randomized into one of these three groups: web-based PA intervention, print-based PA intervention, or WLCG. Further details are outlined in the flow chart in Figure 3.

### Procedure and Interventions

The *PA interventions* in this study were developed on the basis of the self-regulation theory [56,57]; behavior change techniques (ie, goal setting, planning, social support, and feedback) [58]; and (in PROMOTE 2) in a cocreative process with individuals of the target group and stakeholders, such as exercise instructors, leaders of older adults’ facilities, and managers of older adults’ homes. The intervention aimed to improve overall self-monitoring capabilities regarding PA and enable transfer for the time after the intervention.

According to the PA recommendations of the WHO and the American College of Sports Medicine [13], older adults randomized to the IGs were advised to engage in physical exercises. The recommendations included suggestions to improve balance (twice per week), flexibility (twice per week), and strength (twice per week for the 8 major muscle groups). In addition, the participants were instructed to engage in at least 150 minutes per week of moderate endurance training or 75 minutes per week of vigorous training or a combination of both moderate and vigorous training intensities [59].

After randomization, a baseline assessment (time point 0 [T0]) was conducted. Following T0, the IGs in PROMOTE 1 received a *print-based intervention* in the form of a booklet, which was tailored to the individual baseline PA levels. Feedback was tailored to the baseline motivational stage (nonintention, intention, or action) to engage in the recommended PA. In addition, the material was tailored based on sex: pictures of men for male participants and pictures of women for female participants were used to model the recommended exercises. The web-based materials offered in the corresponding study arms in PROMOTE 1 and 2 included access to web-based materials that contained and displayed the same information on exercises for balance, flexibility, endurance, and strength, as for the print-based version. In addition, for PROMOTE 2, a print-based PA diary was developed in the form of an expert-driven approach and contained all exercises; provided the option to enter data on performed exercises; and, thus, visualize personal progress. The web-based intervention from PROMOTE 1 was adapted based on feedback obtained during the first study period. An additional web-based option (PA tracker) to capture the daily step count was included for the randomly selected subgroups in both study arms (PROMOTE 1 and 2).

All IGs were encouraged to use the material and engage in the recommended PA over 10 weeks. These were accompanied by weekly group meetings conducted and moderated by trained staff members. During these meetings, questions concerning the program could be raised. In addition, theoretical inputs for healthy aging were provided. Moreover, physical exercises were performed together with feedback from the participants regarding their exercise practice. At the same time, social interactions among the participants and their contacts with the study team were facilitated. After 10 weeks of group meetings, a follow-up assessment for time point 1 (T1) was conducted 12 weeks after the baseline assessment. Several collected variables served as the basis for this study. More information on the interventions and procedures can be found in previous publications [49-51,53].

### Used Instruments

Adherence was measured according to the WHO recommendations of ≥150 minutes per week of MVPA in bouts of at least 10 minutes. The daily minutes for PA in terms of MVPA were assessed by asking the study participants what activities they performed in bouts of at least 10 minutes. Minutes per week for MVPA in the bouts were derived by multiplying the daily average minutes in the 10-minute bouts by 7. Subsequently, this measure was dichotomized as meeting or not meeting the WHO recommendations. This resulted in a dichotomous variable with 1=does not meet 150 minutes of MVPA recommendation and 2=meets 150 minutes of MVPA at T0 and T1. To determine adherence over time, a categorical variable regarding the change in meeting the recommended 150 minutes of MVPA was computed by subtracting the baseline value from the 12-week follow-up value. The resulting variable indicated whether study participants *remained active or inactive* (0), fell back into *not meeting the recommendation anymore* (−1), or *became active* (1).

To assess the *stage of PA behavior*, participants were asked whether they had performed ≥150 minutes of endurance exercise per week (eg, fast walking, walking, biking, and swimming) at 2 measurement time points (T0 and T1). Participants were asked to respond on a 5-point Likert scale: 1=“No, and I do not intend to start” (precontemplation stage or nonintenders); 2=“No, but I am considering it” (contemplation stage or nonintenders); 3=“No, but I seriously intend to start” (preparation stage or intenders); 4=“Yes, but only for a brief period of time” (action stage or actors); and 5=“Yes, and for a long period of time” (maintenance stage or actors).

The stage item is based on items used by Lippke et al [60]. For this study, participants in the precontemplation and contemplation stages were categorized as nonintenders, participants in the preparation stage were categorized as intenders, and participants in the action and maintenance stage were categorized as actors.

*Intention to engage in regular endurance and strength training* was assessed with 2 items (“I intend to engage in strenuous endurance training for at least 75 minutes per week and strength- and balance training twice a week” and “I intend to engage in moderate endurance training for at least 150 minutes per week and strength- and balance training twice a week”).

These items were based on previous literature [60,61]. Both items were measured at T0 and T1 on a 7-point Likert scale ranging from 1=*completely disagree* to 7=*completely agree*. The 2 items were kept separate as they had discriminant validity (Spearman ρ at T0=0.410; Spearman ρ at T1=0.467; *P*<.001). The retest reliability was Spearman ρ T0 to T1 of 0.531 and 0.378, respectively (*P*<.001).

*Outcome expectancies*, as suggested by Lippke et al [62] and Schwarzer et al [63], were measured using 4 items in total at 2 measurement time points. A total of 2 items measured positive outcome expectancies (“If I engage in 150 minutes of moderately strenuous or 75 minutes of strenuous endurance exercise of strength and balance training twice per week, it is good for my health.” and “If I engage in 150 minutes of moderately strenuous or 75 minutes of strenuous endurance exercise of strength and balance training twice per week, it makes me feel better afterwards.”).

The remaining 2 items focused on negative outcome expectancies (“If I engage in 150 minutes of moderately strenuous or 75 minutes of strenuous endurance exercise of strength and balance training twice per week, it takes too long.” “If I engage in 150 minutes of moderately strenuous or 75 minutes of strenuous endurance exercise of strength and balance training twice per week, it is too costly.”). All 4 items were measured on a 7-point Likert scale ranging from 1=*completely disagree* to 7=*completely agree*.

The 2 items measuring positive outcome expectancies were kept separate as they had rather discriminant validity (Spearman ρ at T0=0.693; Spearman ρ at T1=0.703; *P*<.001). The retest reliability was Spearman ρ T0 to T1 of 0.425 and 0.508, respectively (*P*<.001). The 2 items measuring negative outcome expectancies were also kept separate as they had rather discriminant validity (Spearman ρ at T0=0.474; Spearman ρ at T1=0.443; *P*<.001). The retest reliability was Spearman ρ T0 to T1 of 0.339 and 0.441, respectively (*P*<.001).

*Self-efficacy* was measured with 5 items, in total, at both measurement time points T0 and T1 [61,64]. A single item was used to assess task self-efficacy (“I am confident that I can engage in 150 minutes of moderately strenuous or 75 minutes of strenuous endurance exercise and strength and balance training twice a week, even if it gets difficult.”).

A total of 2 items assessed maintenance self-efficacy (“I am confident that I can engage in 150 minutes of moderately strenuous or 75 minutes of strenuous endurance exercise and strength and balance training twice a week, even if it takes long, until it is a habit.” and “I am confident that I can engage in 150 minutes of moderately strenuous or 75 minutes of strenuous endurance exercise and strength and balance training twice a week, even if I am worried or face problems, e.g., scheduling difficulties.”).

In addition, recovery self-efficacy was measured by 2 items (“I am confident that I can engage in 150 minutes of moderately strenuous or 75 minutes of strenuous endurance exercise and strength and balance training twice a week, even if I postponed my plans several times.” and “I am confident that I can engage in 150 minutes of moderately strenuous or 75 minutes of strenuous endurance exercise and strength and balance training twice a week, even If I suspended several times.”).

All 5 items were measured on a 7-point Likert scale ranging from 1=*completely disagree* to 7=*completely agree*. For this study, the sum of all 5 items was calculated (Cronbach α at T0=.884; Cronbach α at T1=.897). The retest reliability was Spearman ρ at T0 to T1 of 0.475 (*P*<.001).

To further assess social-cognitive predictors, *planning* was measured using 6 items. The items were adapted for this study from those used in previous studies on PA [65].

Three items measured *action planning*: “For the next month, I have already planned where I will be physically active,” “For the next month, I have already planned how I will be physically active,” and “For the next month, I have already planned when and how often I will be physically active.”.

Furthermore, three items assessed the construct of *coping planning*, respectively: “For the next month, I have already planned when I have to take care not to suspend,” “For the next month, I have already planned what I can do in difficult situations to stick to my intentions,” and “For the next month, I have already planned how I can remain physically active even if there are barriers.”

All 6 items were measured on a 7-point Likert scale ranging from 1=*completely disagree* to 7=*completely agree*. For this study, the sum of all 6 items was calculated (Cronbach α at T0=.932; Cronbach α at T1.899). The retest reliability was Spearman ρ T0 to T1 of 0.492 (*P*<.001).

*Habits* were measured using two items at two measurement time points [66]: “Engaging in the recommended endurance, strength and balance training is something that has become my habit.” and “Engaging in the recommended endurance, strength, and balance training is something that I do without thinking about it.”

Both items were measured on a 7-point Likert scale from 1 *completely disagree* to 7 *completely agree* (Spearman ρ at T0=0.474; Spearman ρ at T1=0.443; *P*<.001). The retest reliability was Spearman ρ T0 to T1 of 0.339 and 0.441, respectively (*P*<.001).

Symptoms of *depression* were measured using the CES-D [67] scale both at T0 and T1. The scale comprises 20 items with a possible sum score range of 0 to 60. Each item was measured on a 5-point Likert scale ranging from 0=*rarely or never (<1 day)* to 1=*some or little of the time (1-2 days),* 2=*often or a moderate amount of time (3-4 days)*, and 3=*most of the time (5-7 days)*.

For the purpose of analysis, the mean score of all 20 items was calculated for all participants (Cronbach α at T0=.605, Cronbach α at T1=.587). The retest reliability was Spearman ρ T0 to T1 of 0.759 (*P*<.001).

To assess perceived *loneliness* at T0 and T1, the item *I felt lonely* was used from the CES-D scale [67]. The item was measured on a 4-point Likert scale from 0=*rarely or never (<1 day)* to 1=*some or little of the time (1-2 days)*, 2=*often or a moderate amount of time (3-4 days)*, and 3=*most of the time (5-7 days)*. The retest reliability was Spearman ρ T0 to T1 of 0.597 (*P*<.001).

*Sociodemographic* data were collected using a questionnaire administered before the intervention (at the baseline level). The questionnaire was formulated according to the German Health Interview and Examination Survey for Adults [68]. The collected variables included date of birth, sex of the participants (male or female), height (in cm), and weight (in kg).

In addition to the date of birth, *perceived age* was measured using an open-ended question. The participants were asked, “How old do you feel?” Perceived age was assessed at T0 and T1. The retest reliability was Spearman ρ T0 to T1 of 0.826 (*P*<.001). Furthermore, country of birth, mother tongue, family status, living alone, number of children, qualification, educational level, and employment status were assessed.

Employment status was measured with a single item taken from a questionnaire assessing demographic and sociostructural data from German older adults and adapted for this study [69]. Qualification and educational level were measured with 2 items and aggregated based on the 2016 version (volume 17) of the International Standard of Education [70].

BMI was calculated using height and weight and categorized into underweight, normal weight, overweight, and obese, according to the WHO BMI classification for adults aged ≥20 years [71].

All the used instruments were validated before and are described in the study protocols [49,51], as well as in previous publications [50,52-54].

### Analysis Sample

IG allocations from PROMOTE 1 and 2 were summarized within a pooled IG variable that included the following three categories: *a*=WLCG from PROMOTE 1 (reference), *b*=web-based IG from PROMOTE 1 and 2, and *c*=print-based IG from PROMOTE 2.

Only the participants who completed the baseline assessment (T0) were included in the analysis. In PROMOTE 1, participants’ cognitive status was assessed using the MMSE [72] 1 week before the start of the intervention phase. In PROMOTE 2, participants’ cognitive status was assessed using MMSE-2:BV [73] during the first weekly group meeting (ie, the start of the intervention phase). Participants who scored <25 points on the MMSE or <13 points on the MMSE-2:BV were excluded from the analysis. Amendments to the cutoff values for exclusion because of cognitive impairment have been discussed in previous publications [50,52].

After excluding individuals with cognitive impairment (37/831, 4.5%; Figure 3) and missing information on baseline demographic characteristics (73/831, 8.8%), the analysis sample included 721 older adults (see *Data Exclusion* section and Figure 3). To determine adherence, a variable regarding meeting the recommended 150 minutes of MVPA was computed by subtracting the baseline measure from the T1 measure. The resulting variable thereby indicated whether study participants *remained active or inactive* (0), fell back into *not meeting recommendations anymore* (−1), or *became active* (1).

### Statistical Analysis

#### Preparation

All analyses were performed using SPSS (version 27; IBM Corp). The Little missing completely at random test (*P*>.05) suggested that data were not missing completely at random (ie, it suggested that data were missing systematically). Assuming that existing data could be used to produce an estimate of the missing information (ie, assuming that data were missing at random) [74], single data imputation was implemented by using the expectation-maximization algorithm.

#### Test of Hypotheses

To assess whether the 2 IGs outperformed the WLCG (hypothesis 1) on self-reported behavior and stage of change, frequency analyses and chi-square tests, *Z* tests, and contingency coefficient tests were used to test the number of participants who adopted or maintained an active lifestyle.

Changes in social-cognitive predictors (hypothesis 2) were analyzed with repeated-measures multivariate analysis of variance (MANOVA) via mixed-effects generalized linear models with group and time as factors. For the *F* values, the Roy largest root was reported.

The primary aim of the moderated mediation analyses was to investigate whether the IG allocation (independent variable or predictor) had an effect on perceived symptoms of depression (CES-D score) at T1 (dependent variable or outcome), which is mediated by subjective age at T1 and moderated by the stage of change in endurance training at T0 (hypothesis 3; see Figure 1 for the proposed model).

The secondary aim of the moderated mediation analysis was to investigate the same relationship with loneliness as the dependent variable (hypothesis 4; see Figure 2 for the proposed model). These associations were investigated using moderated mediation models within the PROCESS macro (version 3.0; Hayes, The International Association of Applied Psychology mediation analysis).

The models were adjusted for the following baseline variables: loneliness and CES-D score, as well as subjective age, sex, age, educational status (International Standard of Education), family status, and BMI (all at T0). A bootstrapping approach of 10,000 samples and a specific seed (seed=1) was applied to ensure robust and replicable results. The effect sizes were represented by unstandardized regression coefficients. To calculate the heteroscedasticity-robust SE, the HC3-Option in the process function was used. Accordingly, the assumption of homoscedasticity could be avoided.

#### Data Exclusion

The analyses were conducted following the intention to treat principle; that is, participants were included in primary analyses according to their original group allocation and disregarding study completion. This was managed by missing value imputation using an expectation-maximization algorithm.

In addition, according to the study protocol, participants with cognitive impairments were excluded (37/831, 4.5%; Figure 3). In addition, missing baseline demographic information was not imputed; thus, participants with missing information on sex, age, educational status, family status, or BMI were excluded from the analyses (73/831, 9%).

## Results

### Hypothesis 1

To test whether the 2 interventions outperformed the WLCG (hypothesis 1), the study participants who adopted or maintained an active lifestyle were analyzed, as recommended by the WHO. First, those who did *not meet the recommendation regarding PA at T0* based on self-reported adherence were investigated; of those individuals, more individuals became adherent if they were exposed to the web-based intervention (47/59, 80%; Table 1) or received the print-based intervention (20/25, 80%) than those who were not treated (WLCG; 5/7, 71%). At a descriptive level, the numbers indicate the favoring of the IG over the control group.

Second, those who *met the recommendation regarding PA before the study* were investigated; of these, 600 individuals self-reported to be adherent at T1. More individuals remained adherent if they were exposed to the web-based intervention (396/411, 96.4%) or not treated (WLCG; 141/150, 94%) than those receiving the print-based intervention (63/69, 91%).

The difference in the proportion of adopters between the web-based and print-based interventions was statistically significant (*Z*=-1.94; *P*=.02); as well as the differences between the IGs and the WLCG were significant (WLCG vs print *Z*=2.3967 and WLCG vs web *Z*=3.8367; both *P*=.01).

This finding was replicated by the stages of change in endurance training. In Table 2, the number of study participants in the 3 intervention conditions moving from the nonintenders, intenders, or actor stage to another stage or remaining in the former stage is reported.

In all stage groups, the percentage of individuals moving a stage forward (from nonintentional stage to intentional or action, and from intentional to action) or maintaining the stage when starting as an actor was higher in the web-based or print group than in the WLCG (Table 2). In contrast, in the WLCG, the percentage of individuals remaining in the nonintentional or intentional stage was higher than that in the IGs (Table 2). The pattern in Table 2 was statistically significant (*χ*^2^_4_=79.1; *P*<.001; contingency coefficient 0.314; *P*<.001). *Z* tests were performed to test whether group differences in the stage of change movements were statistically significant, which was the case for initial intenders who moved to the actor stage (WLCG vs web-based *Z*=−4.2325; *P*=.01 and WLCG vs print-based *Z*=−5.349; *P*=.01) and initial actors who remained in the actor stage (WLCG vs print *Z*=−3.1853; *P*<.01).

Summarizing the findings regarding hypothesis 1 that the web-based and print-based interventions outperformed the control condition in terms of PA behavior change, we can conclude that our results suggest this direction. The web-based intervention seemed to work better in terms of the prevention of remaining in or a relapse into *not meeting recommendation*s (Table 1) and adopting or remaining in the intender or actor stage (Table 2).

**Table 1:** Numbers and percentages of study participants assigned to 1 of 3 experimental groups regarding who met or did not meet the recommended physical activity level at T0^a^ and T1^b^.

At T0		At T1, n (%)			Total, N
		Not meeting the recommendation	Meeting the recommendation		
**Not meeting the recommendation**					
	WLCG^c^	2 (28.6)	5 (71.4)	7	
	Web-based	12 (20.3)	47 (79.7)	59	
	Print-based	5 (20)	20 (80)	25	
	Total	19 (20.9)	72 (79.1)	91	
**Meeting the recommendation**					
	WLCG	9 (6)	141 (94)	150	
	Web-based	15 (3.6)	396 (96.4)	411	
	Print-based	6 (8.7)	63 (91.3)	69	
	Total	30 (4.8)	600 (95.2)	630	

^a^T0: time point 0.

^b^T1: time point 1.

^c^WLCG: wait-list control group.

**Table 2:** Cross-tabulation of nonintenders, intenders, and actors at T0^a^ moving to or remaining nonintenders, intenders, and actors at T1^b^ depending on the experimental group they were in.

T0 and T1			Nonintenders, n (%)		Intenders, n (%)		Actors, n (%)		Total, N
**Nonintenders**									
	WLCG^c^	36 (59)		16 (26.2)		9 (14.8)		61	
	Web-based	35 (16.7)		46 (22)		128 (61.2)		209	
	Print-based	9 (17.6)		13 (25.5)		29 (56.9)		51	
**Intenders**									
	WLCG	7 (23.3)		11 (36.7)		12 (40)		30	
	Web-based	5 (4.8)		11 (10.6)		88 (84.6)		104	
	Print-based	4 (16)		2 (8)		19 (76)		25	
**Actors**									
	WLCG	16 (24.2)		8 (12.1)		42 (63.6)		66	
	Web-based	9 (5.7)		7 (4.5)		141 (89.8)		157	
	Print-based	2 (11.1)		0 (0)		16 (88.9)		18	
**All stages together**									
	WLCG	59 (37.6)		35 (22.3)		63 (40.1)		157	
	Web-based	49 (10.4)		64 (13.6)		357 (76)		470	
	Print-based	15 (16)		15 (16)		64 (68.1)		94	
	Total	123 (17.1)		114 (15.8)		484 (67.1)		721	

^a^T0: time point 0.

^b^T1: time point 1.

^c^WLCG: wait-list control group.

### Hypothesis 2

A total of 2 MANOVAs with 19 predictors (Multimedia Appendix 1, Table S1) and 3 outcomes (Multimedia Appendix 1, Table S2) were calculated to test hypothesis 2.

The first MANOVA revealed a significant difference between the 3 groups, favoring the 2 IGs over and above the WLCG (*F*_19, 701_=4.778; *P*<.001; *η*^2^=0.098), as well as a significant interaction between time and group (*F*_19, 701_=2.778; *P*<.001; *η*^2^=0.070).

**Figure 4:**
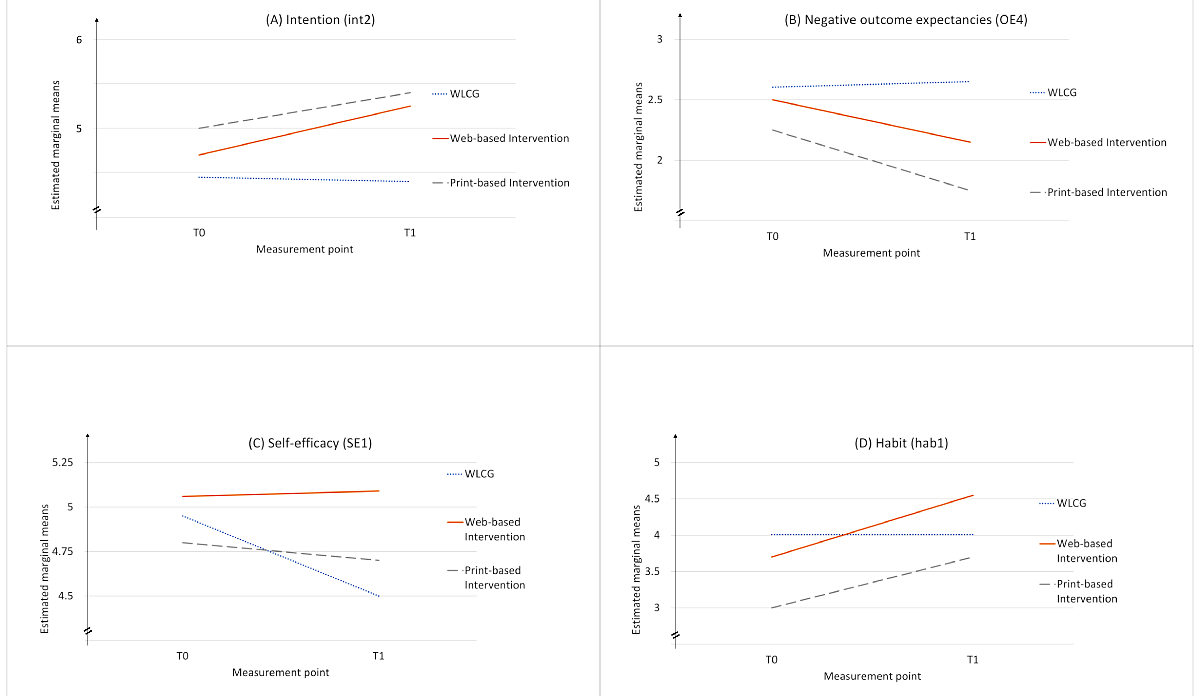
(A) Intention, (B) negative outcome expectancies, (C) self-efficacy, and (D) habit. T0: time point 0; T1: time point 1; WLCG: wait-list control group.

This effect was mainly based on intention, negative outcome expectancies, planning, and habit (see Multimedia Appendix 1, Table S1, for the means, SDs, and statistics). Figure 4 outlines the development, indicating that the WLCG dropped slightly with its intention over time, whereas the 2 IGs improved over time. Figure 4 shows that the WLCG retained its negative outcome expectancies, whereas the 2 IGs improved in terms of perceiving fewer negative outcomes. Figure 4 also demonstrates that the WLCG dropped with its self-efficacy, whereas self-efficacy remained stable in the web-based IG and increased in the print-based IG. Finally, Figure 4 shows that the WLCG remained stable in terms of habit strength, whereas the 2 IGs improved over time.

With the second MANOVA testing the outcomes, the effects of the interventions on subjective aging, loneliness, and symptoms of depression were tested (Multimedia Appendix 1, Table S2). Both the between-group effect (*F*_3, 717_=8.668; *P*<.001; *η*^2^=0.018) and the interaction of group and time were significant (*F*_3, 717_=6.101; *P*<.001; *η*^2^=0.025). The group effect was mainly based on subjective age and symptoms of depression, and the interaction effect was based on symptoms of depression with regard to group and time on loneliness (see Multimedia Appendix 2, Table S1-S3 with means, SDs, and statistics).

**Figure 5:**
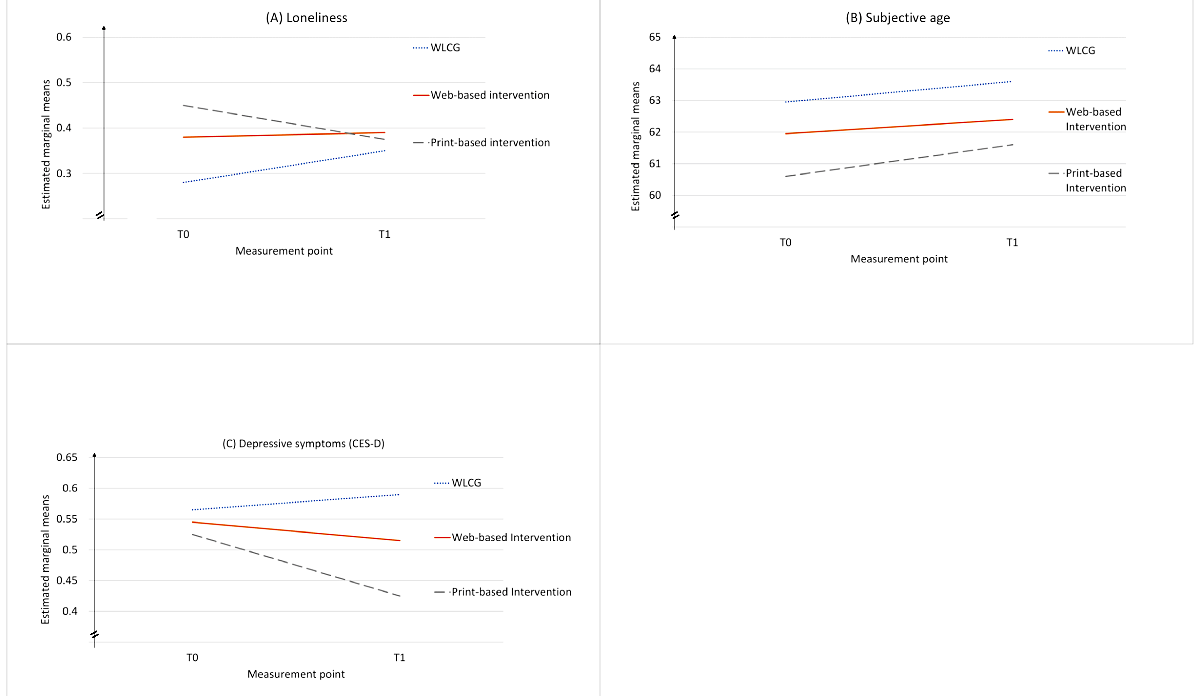
(A) Loneliness, (B) subjective age, and (C) symptoms of depression. CES-D: Center for Epidemiologic Studies Depression; T0: time point 0; T1: time point 1; WLCG: wait-list control group.

Figure 5 highlights that the effect of *loneliness* comes from regression to the mean, with the WLCG increasing in its loneliness, the web-based IG maintaining its previous level, and the print-based IG decreasing in its loneliness over time. With *subjective age*, all groups showed an increase over time (Figure 5). Differences from baseline values remained. Figure 5 also shows that all groups started off at almost the same level of depressive symptoms. However, over time, the WLCG increased with regard to the reported symptoms of depression, whereas the 2 IGs decreased, with an even better effect of the print-based intervention than that of the web-based intervention.

Overall, the effect sizes were rather small, ranging from *η*^2^=0.098 to 0.018.

Summarizing the findings regarding hypothesis 2, we found overall support. The web-based and print-based interventions improved the social-cognitive predictors of PA behavior change, subjective aging, loneliness, and depression compared with the control condition. The web-based and print-based interventions were significantly different from the control condition.

### Hypothesis 3

**Figure 6:**
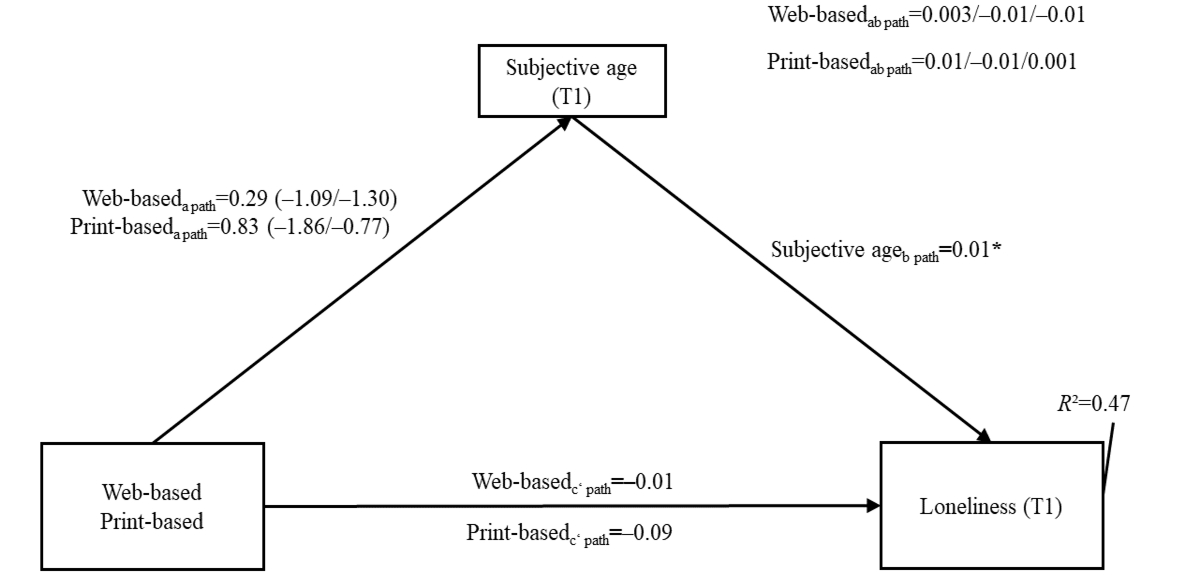
Moderated mediation model results for loneliness. The interaction between the intervention group and the stage of change in the a path is shown in parentheses. The first value represents the intention stage, and the second value represents the actor stage. The moderation of the ab path is shown on the upper right. The first value represents the nonintenders stage, the second value represents the intenders stage, and the third value represents the actor stage. The model was adjusted for the following baseline variables: loneliness, subjective age, sex, age, educational status (International Standard Classification of Education), family status, and BMI (all at time point 0). The wait-list control group was used as a reference. *Statistically significant value. T1: time point 1.

To test the mechanisms and hypothesis 3, we conducted a moderated mediation analysis. The results are shown in Figure 6 and Tables S1 and S2 in Multimedia Appendix 2.

None of the interventions had a significant direct effect on loneliness compared with the WLCG (web-based: *c*′ path −0.01, 95% CI −0.11 to 0.08, SE 0.05; print-based: *c*′ path −0.09, 95% CI −0.24 to 0.06, SE 0.08). Regarding the mediator subjective age (subjective age), a significant positive relationship between subjective age and loneliness at T1 was found (subjective age: *b* path 0.01, 95% CI 0.001-0.02; SE 0.01). However, the effect size was very small, and no significant indirect effects of the interventions on loneliness through subjective age were revealed. To summarize, the results suggest that subjective age could not account for a significant proportion of the relationship between the IGs and loneliness. Furthermore, there was no significant interaction, suggesting that there was no moderation of the stage of change in endurance activities.

### Hypothesis 4

To test hypothesis 4, another moderated mediation model was tested.

**Figure 7:**
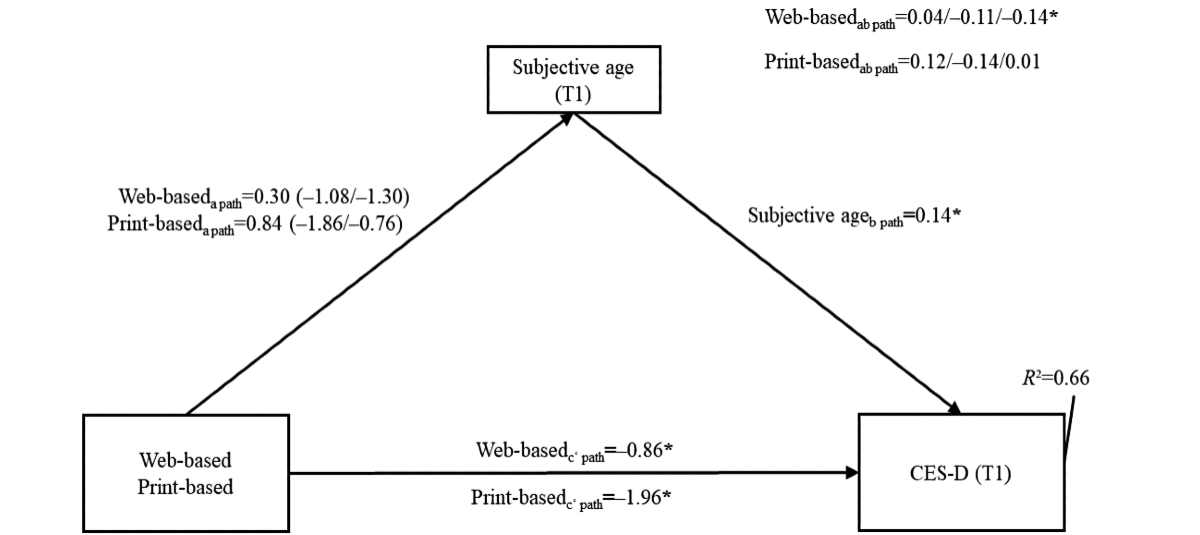
Moderated mediation model results for depression. The interaction between the intervention group and the stage of change in the a path is shown in brackets. The first value represents the intention stage, and the second value represents the actor stage. The moderation of the ab path is shown in the upper right. The first value represents the nonintenders stage, the second value represents the intenders stage, and the third value represents the actor stage. The model was adjusted for the following baseline variable: depressive symptoms (CES-D), subjective age, sex, age, educational status (International Standard Classification of Education), family status, and BMI (all at time point 0). The wait-list control group was used as a reference. *Statistically significant values. CES-D: Center for Epidemiologic Studies Depression; T1: time point 1.

The results are shown in Figure 7 and Tables S3 and S4 in Multimedia Appendix 2.

Validating the previous analyses, both interventions had a significant direct effect on the symptoms of depression at T1 compared with the WLCG (web-based: *c*′ path −0.86, 95% CI −1.58 to −0.13, SE 0.38; print-based: *c*′ path −1.96, 95% CI −2.99 to −0.92, SE 0.53). Furthermore, subjective age at T1 was positively related to depressive symptoms (subjective age: *b* path 0.14, 95% CI 0.05-0.23; SE 0.05). An indirect relationship between the intervention and the symptoms of depression via subjective age was only present for participants who were both in the actor stage of change for endurance activities and received the web-based intervention (web-based: *ab* path −0.14, 95% CI −0.34 to −0.01; SE 0.09).

Summarizing the findings regarding hypothesis 4, only older adults in the actor stage of endurance training who received the web-based intervention were associated with lower symptoms of depression at T1, which was partially mediated by subjective age at T1. Thus, the existence of moderated mediation was confirmed, although all other mediation pathways were not significant, and the effect sizes were small.

## Discussion

### Principal Findings

This study aimed to compare the effects of web- and print-based PA interventions on self-reported PA, stage of change, determinants of PA, loneliness, and depression. Moreover, the goal was to investigate whether subjective age is a mediator and whether the stage of change is a moderator of the effectiveness in 831 older individuals participating in the PROMOTE 1 or PROMOTE 2 study.

The main finding was that the print-based and web-based interventions both worked well and helped a higher proportion of individuals meet the recommendations for PA and move forward with their stage of change compared with the WLCG. Support for the effectiveness of the interventions was also found regarding the social-cognitive predictors of PA behavior. None of the interventions had a significant direct effect on loneliness compared with the control group. Thus, the main assumption that a PA intervention always helps reduce loneliness does not hold true. It seems more important to take mastery experience into account: the results of our moderated mediation analyses suggest that compared with the WLCG, receiving the web-based intervention was associated with lower symptoms of depression at T1 and that subjective age could explain a substantial proportion of variance. However, this holds true only for participants in the actor stage of change for endurance activities. The mechanisms are in accordance with the assumption that mastery experience and self-regulation—operationalized with subjective age—help the study participants who are already physically active at the baseline to reduce their symptoms because of maintained or improved physical perceptions and repairing or maintaining interpersonal skills and relationships [6]. In this group, the PA IG allocation reduced the likelihood of depressive symptoms and loneliness if they actually felt younger.

However, compared with the wait-list control, the intervention did not help older adults feel less lonely, perhaps because of 2 aspects. We revealed a floor effect (ie, generally low loneliness levels). Moreover, one should also keep in mind that the intervention was not designed to reduce loneliness but to increase PA; accordingly, the relationship between the intervention and feeling lonely was not strong enough to be of statistical importance. Nevertheless, the effects in the IGs underlined the importance of supporting active older adults to remain physically active to feel fit and subjectively young, as well as to lower symptoms of depression. In the following paragraphs, the hypotheses are reviewed in more detail, followed by more discussion.

Hypothesis 1, assuming that web-based and print-based interventions outperform the control condition in terms of self-reported PA behavior change, was confirmed by our data. The interventions seemed to work better in terms of preventing a relapse into *not meeting recommendations anymore* and moving study participants into the actor stage. Without any intervention, 71% (5/7) of the previously inactive participants became active at the recommended level. This percentage was higher in the web-based (47/59, 80%) and print-based groups (20/25, 80%). Without any intervention, 94% (141/150) of the previously active study participants remained active at the recommended level. This percentage was slightly higher in the web-based group (396/411, 94%).

When replicating this finding with the stages of behavior change, the effects clearly demonstrated the benefits of the interventions; in the untreated WLCG, individuals were more likely to remain in or relapse to the nonintentional stage than the individuals in the 2 IGs.

Our results also support hypothesis 2: participation in web-based and print-based PA interventions was associated with improvements in the social-cognitive predictors of PA behavior change, self-reported PA behavior change, subjective age, loneliness, and depressive symptoms compared with the WLCG. The web-based and print-based interventions were significantly different from the WLCG, which matched expectations.

Hypothesis 3 was not supported by our results, as we found that the results were not consistent with the assumption that individuals in the IG would benefit from fewer feelings of loneliness as they would experience a decrease in subjective age. Regarding hypothesis 4, only older adults who were in the actor stage regarding endurance training and who received the web-based intervention revealed an intervention effect on depressive symptoms (CES-D score), partially mediated by subjective age at T1. This subgroup reported a lower subjective age than that of the WLCG, which was further associated with reporting lower symptoms of depression at T1.

### Limitations and Suggestions for Future Work

In this study, only subjective data were included as most of the variables such as social-cognitive variables, stage of change, loneliness, subjective age, and symptoms of depression could only be measured in this form. However, the behavior should be assessed using objective measures (such as an activity tracker). Accordingly, validation studies are required to better understand behavior changes more thoroughly.

In addition, the included study participants were not representative samples, as many individuals had already met the behavioral criteria, especially in the first study phase (PROMOTE 1). Despite the adaptation of the inclusion criteria for the second phase of the study, the target group of individuals with low activity was still not sufficiently reached, and their needs were not adequately addressed. This is a common problem in public health intervention research, which requires further effort (eg, to improve the recruitment of nonactive and low-motivation individuals). Therefore, future studies should attempt to overcome this problem. Therefore, to address the needs of these individuals more effectively, need-based assessments (ie, tailored to current circumstances and hindering factors) should be conducted in future studies.

Another limitation is access to, and availability of, web-based technology, particularly among the studied age groups. Thus, in this context, on the one hand, some of the respondents interested in the study invitation had to be excluded from participation as they did not meet the inclusion criterion of owning a PC or did not have access to the internet.

On the other hand, personal affinity for and acceptance of modern technology services, which in part becomes apparent over the course of the study, might affect the success of information technology interventions and, therefore, should not be overlooked. These eligibility criteria partly exclude disadvantaged groups with different psychological preconditions and developmental possibilities. In the future, such limitations should be overcome by making study participation more accessible to individuals with low computer or internet literacy and technology affinity.

Nevertheless, although it is helpful to compare rather *conventional* print alternatives with web-based interventions, it should also be considered that future senior populations are likely to have more digital competence. Web-based interventions offer inclusionary benefits that can be of particular interest when dealing with an older adult population (eg, text-to-speech options and variable font sizes). Therefore, the design, content, formatting, and acceptance of digital or internet interventions should be further researched and tailored when implementing interventions for older adults.

In addition, a critical point is selective dropout (eg, higher attrition in either the web-based IG, in the groups that received only print-based material, or the WLCG). This was particularly evident in the first phase of the study among the groups with high technology requirements (web material and activity trackers that had to be synchronized with the website). Accordingly, future projects should support those at risk of dropping out of the intervention to remain in the study.

A further shortcoming of this study is that the interventions were designed to improve PA and did not explicitly reduce loneliness or symptoms of depression directly. As the findings are promising, future studies should follow up on this as PA can also be a very effective tool to address these emotions and cognitions. Parts of the data have already been published, such as the effects of the interventions on the stages of change in PROMOTE 1 [50,53]. Consequently, when using these data for a systematic review or meta-analysis, this needs to be taken into account.

In general, whether older adults benefit from improved health (other indicators in addition to symptoms of depression) or well-being (other indicators in addition to loneliness), health behavior, and its predictors from print-based material or web-based interventions still requires more attention, and future studies should follow up on the mechanisms that explain how the interventions work.

### Process Analysis

The results of the moderated mediation analysis revealed that there is not only a simple linear effect of the intervention on symptoms of depression but also that the effects of the intervention on symptoms of depression are modeled in a more complex way. Therefore, it can be hypothesized that interventions generally tend to affect target variables in a nonlinear fashion and that, in general, interventions and health should be understood as complex systems. With the actor stage as a moderator, the conceivable conclusion is that only people who were physically active before the intervention benefited from it with regard to the symptoms of depression. Therefore, the HAPA theory is a helpful tool in designing interventions as it can assist in identifying a person’s current stage of change and aid them in moving to the actor stage.

Li et al [47] found that subjective age is related to various health benefits. In their study, subjective age acted as a mediator between the intervention and symptoms of depression. With this in mind, the following can be assumed: in interventions, a set of variables that can act as potential mediators exists, as these variables are linked to several health benefits. However, it is easy to overlook these mediators when only focusing on linear and bivariate effects, when, in fact, a mediation might only apply to a subgroup of individuals. Future research needs to identify this set of variables and implement it effectively in intervention frameworks. Subjective age might be one of these variables, although there might be more underlying components.

### Comparison With Prior Work

Loneliness and mental health in the aging population are important topics, and our data match previous work showing that physical exercise is a key factor in addressing these issues [4,6]. However, the effects of the intervention were stronger regarding behavior adherence and stage (contingency coefficient 0.314), as well as behavioral habits (*η*^2^=0.025-0.026), than regarding loneliness and depression (*η*^2^=0.005-0.024). The finding of stage-specific effects (only actors benefited) with regard to depression matches the previous finding that interventions improving PA can also improve mood [7].

To date, little research has been conducted on whether web-based or print-based interventions are more likely to result in successful behavior change regarding PA, which can simultaneously affect feelings of loneliness and depression. By matching the findings of Golsteijn et al [9], this study revealed some benefits of the print-based intervention for this age group [9]. However, in general, the web-based and print-based interventions were more effective than the WLCG.

Aspects assumed to affect participation in, and effectiveness of, interventions have been previously studied. With respect to the uptake of PA offerings and intervention modalities, sociodemographic differences have emerged in the past. Accordingly, women, those with higher levels of education, and those who are already physically active are more likely to participate [23,75]. Physical inactivity, being overweight, and having a low educational status were indicators of discontinuation of the intervention before it was completed [22]. Furthermore, it is known that younger individuals seem to prefer web-based services [8,76], whereas older adults or women appear more likely to favor print-based offerings [76].

This and former analyses of the PROMOTE 1 study [50] identified higher attrition rates in the IGs than in WLCG, which is in line with other similar intervention studies [77]. This may be explained by the fact that the participants were more motivated to stay in the study as they received the program afterward. However, those who obtained the intervention later also predominantly maintained their behavior over the course of the intervention. Cunningham et al [78] found that wait-list groups interrupt efforts to change and pointed to the importance of activities toward changing different psychological preconditions and developmental possibilities. Therefore, to reduce the dropout rate in the WLCG, individuals randomized to this group should be informed about the progress and expected time of participation. As previous studies have indicated that longer waiting times are associated with higher dropout rates, the study design should be adapted to decrease the waiting time for the WLCG [8,22,54].

In addition, in the first phase of the study, the problem of increased dropout or greater dissatisfaction in the study arms with a need for greater technical skills and information technology acceptance became relevant [50]. This has also been reported by other authors [79]. As a reaction, the study design for PROMOTE 2 was adapted such that participants could change the type of materials provided at a defined time point on a preference basis, which could improve the loss of participants because of this aspect, as well as the satisfaction with the intervention in a recognizable way [52].

### Conclusions

In times of physical distancing, for instance, during the COVID-19 pandemic, alternative forms of support, such as print- and web-based PA exercise content for individual implementation at home, are essential and in high demand. As users are very heterogeneous, tailoring PA interventions according to their specific needs (including differing motivations to engage in PA) and previous experiences (captured by stages), as well as according to their individual technology-based preconditions, could be an effective approach to initiating behavior change with regard to PA. Particularly, for digital interventions, the varying availability and use preferences of digital devices should be considered.

Tools for individual use, including activity monitoring (such as exercise diaries) and exercise instructions, are highly relevant for location- and time-independent use. Finally, but importantly, the relevance is determined by the reduced mobility in old age and, thus, the possibility or need for exercise at home.

Successful aging in terms of helping older adults feel fit to perform PA should be considered more explicitly, which can help to ensure that a PA intervention actually translates into the reduction of symptoms of depression. At the same time, taking the stage of change-specific aspects into account can benefit the knowledge that, similar to this study, the interventions worked well in intenders and actors for successful aging and symptoms of depression but not in nonintenders. Nonintenders might need other support such as just-in-time adaptive interventions and more instant social support, not only through print-based and web-based modes. However, the delivered interventions appeared to be supportive of intenders and actors and improve the predictors of behavior. Ingredients of the intervention’s behavior change techniques (ie, goal setting, planning, social support, and feedback [58]) paid off.

## Appendix

Multimedia Appendix 1 Effect of the web and print interventions in comparison to the wait-list control group on social-cognitive variables (Table S1), and on loneliness, perceived age and symptoms of depression (Table S2).

Multimedia Appendix 2 Ordinal least squares regression model results for subjective age (Table S1), loneliness (Table S2), subjective age (Table S3) and depressive symptoms (Table S4).

## Abbreviations

CES-D: Center for Epidemiologic Studies Depression
HAPA: Health Action Process Approach
IG: intervention group
MANOVA: multivariate analysis of variance
MMSE: Mini-Mental State Examination
MMSE-2: BV: Mini-Mental State Examination second edition: Brief Version
MVPA: moderate to vigorous physical activity
PA: physical activity
T0: time point 0
T1: time point 1
WHO: World Health Organization
WLCG: wait-list control group
