# Supplementary material for: Mitigating Feelings of Loneliness and Depression by Means of Web-Based or Print-Based Physical Activity Interventions: Pooled Analysis of 2 Community-Based Intervention Trials
Source: JMIR Aging. 2022 Aug 9;5(3):e36515. doi: 10.2196/36515 (PMC9399846; doi:10.2196/36515)
Supplement: Multimedia Appendix 1 [file aging_v5i3e36515_app1.docx]

**Appendix Table S1**. *Effect of the web- and print interventions on social-cognitive variables in comparison to the WLCG: means, standard deviations and statistics*

|  | Experimental group | | | Statistic time (row for T0) and time*experimental Group (row for T1) | |  |
| --- | --- | --- | --- | --- | --- | --- |
|  | WLCG^a^ | Web | Print |  |  |  |
| **Intention 75min** |  |  |  |  |  |  |
| T0 | 4.14 (2.23) | 4.19 (2.12) | 3.98 (2.08) | F(1,837) = 1.365; P = .243; Eta² = .002 |  |  |
| T1 | 3.99 (1.84) | 4.46 (1.87) | 4.06 (1.90) | F(1,837) = 2.936; P = .054; Eta² = .007 |  |  |
| **Intention 150min** |  |  |  |  |  |  |
| T0 | 4.44 (2.17) | 4.70 (2.01) | 4.99 (1.89) | F(1,837) = 6.277; P = .012; Eta² = .007 |  |  |
| T1 | 4.40 (1.82) | 5.23 (1.59) | 5.37 (1.70) | F(1,837) = 5.240; P = .005; Eta² = .012 |  |  |
| **Outcome expectations positive (health)** |  |  |  |  |  |  |
| T0 | 6.11 (1.29) | 6.18 (1.35) | 6.25 (1.31) | F(1,837) = .376; P = .540; Eta² < .001 |  |  |
| T1 | 5.88 (1.48) | 6.23 (1.10) | 6.20 (1.39) | F(1,837) = 1.723; P = .179; Eta² = .004 |  |  |
| **Outcome expectations positive (feel better)** |  |  |  |  |  |  |
| T0 | 5.67 (1.47) | 5.91 (1.48) | 5.87 (1.49) | F(1,837) = .915; P = .339; Eta² < .001 |  |  |
| T1 | 5.52 (1.49) | 5.89 (1.28) | 5.65 (1.45) | F(1,837) = 0.728; P = .483; Eta² = .002 |  |  |
| **Outcome expectations negative (takes long)** |  |  |  |  |  |  |
| T0 | 3.11 (1.92) | 3.19 (1.96) | 3.79 (2.06) | F(1,837) = 7.237; P = .007; Eta² = .009 |  |  |
| T1 | 3.29 (1.85) | 3.01 (1.70) | 3.15 (2.02) | F(1,837) = 6.489; P = .002; Eta² = .015 |  |  |
| **Outcome expectations negative (too costly)** |  |  |  |  |  |  |
| T0 | 2.63 (1.97) | 2.50 (1.89) | 2.26 (1.78) | F(1,837) = 19.168; P < .001; Eta² = .022 |  |  |
| T1 | 2.66 (1.76) | 2.17 (1.55) | 1.74 (1.22) | F(1,837) = 4.850; P = .008; Eta² = .011 |  |  |
| **Self-efficacy (task)** |  |  |  |  |  |  |
| T0 | 4.94 (1.92) | 5.06 (1.81) | 4.81 (1.70) | F(1,837) = 5.415; P = .020; Eta² = .006 |  |  |
| T1 | 4.51 (1.76) | 5.09 (1.55) | 4.72 (1.73) | F(1,837) = 4.009; P = .018; Eta² = .009 |  |  |
| **Self-efficacy (maintenance 1)** |  |  |  |  |  |  |
| T0 | 4.68 (1.84) | 4.67 (1.86) | 4.71 (1.64) | F(1,837) = 4.592; P = .032; Eta² = .005 |  |  |
| T1 | 4.30 (1.76) | 4.58 (1.69) | 4.62 (1.81)s | F(1,837) = 1.535; P = .216; Eta² = .004 |  |  |
| **Self-efficacy (maintenance 2)** |  |  |  |  |  |  |
| T0 | 3.98 (1.79) | 4.07 (1.90) | 4.23 (1.64) | F(1,837) = .365; P = .546; Eta² < .001 |  |  |
| T1 | 3.97 (1.72) | 4.18 (1.66) | 4.25 (1.71) | F(1,837) = .135; P = .873; Eta² < .001 |  |  |
| **Self-efficacy (recovery 1)** |  |  |  |  |  |  |
| T0 | 4.50 (1.90) | 4.71 (1.89) | 4.71 (1.73) | F(1,837) = .135; P = .713; Eta² < .001 |  |  |
| T1 | 4.61 (1.79) | 4.78 (1.68) | 4.60 (1.62) | F(1,837) = .226; P = .798; Eta² = .001 |  |  |
| **Self-efficacy (recovery 2)** |  |  |  |  |  |  |
| T0 | 4.54 (1.88) | 4.67 (1.87) | 4.47 (1.72) | F(1,837) = .559; P = .455; Eta² = .001 |  |  |
| T1 | 4.50 (1.84) | 4.85 (1.68) | 4.44 (1.60) | F(1,837) =.707; P = .494; Eta² = .002 |  |  |
| **Action planning 1** |  |  |  |  |  |  |
| T0 | 5.14 (1.93) | 4.93 (2.26) | 4.38 (2.31) | F(1,837) = 18.847; P < .001; Eta² = .022 |  |  |
| T1 | 5.28 (1.79) | 5.31 (1.77) | 5.12 (1.97) | F(1,837) = 2.186; P = .113; Eta² = .005 |  |  |
| **Action planning 2** |  |  |  |  |  |  |
| T0 | 5.25 (1.84) | 5.01 (2.18) | 4.56 (2.20) | F(1,837) = 13.004; P < .001; Eta² = .022 |  |  |
| T1 | 5.29 (1.76) | 5.41 (1.66) | 5.08 (1.87) | F(1,837) = 2.148; P = .117; Eta² = .005 |  |  |
| **Action planning 3** |  |  |  |  |  |  |
| T0 | 4.98 (1.89) | 4.72 (2.18) | 4.29 (2.15) | F(1,837) = 16.980; P < .001; Eta² = .020 |  |  |
| T1 | 5.05 (1.70) | 5.13 (1.67) | 4.82 (1.93) | F(1,837) = 2.219; P = .120; Eta² = .005 |  |  |
| **Coping planning 1** |  |  |  |  |  |  |
| T0 | 3.99 (2.04) | 3.91 (2.10) | 3.99 (2.08) | F(1,837) = 2.859; P = .091; Eta² = .003 |  |  |
| T1 | 4.05 (1.84) | 4.19 (1.85) | 4.18 (1.99) | F(1,837) =.589; P = .555; Eta² < .001 |  |  |
| **Coping planning 2** |  |  |  |  |  |  |
| T0 | 3.97 (2.01) | 3.76 (2.05) | 3.61 (1.98) | F(1,837) = 1.864; P = .172; Eta² = .002 |  |  |
| T1 | 3.90 (1.81) | 4.10 (1.82) | 3.70 (1.86) | F(1,837) =3.256; P = .039; Eta² = .008 |  |  |
| **Coping planning 3** |  |  |  |  |  |  |
| T0 | 4.42 (1.94) | 4.15 (2.12) | 3.79 (2.04) | F(1,837) = 3.256; P = .072; Eta² = .004 |  |  |
| T1 | 4.37 (1.86) | 4.53 (1.76) | 3.94 (1.96) | F(1,837) = 1.960; P = .141; Eta² = .005 |  |  |
| **Habit 1** |  |  |  |  |  |  |
| T0 | 4.02 (2.23) | 3.71 (2.35) | 2.99 (2.28) | F(1,837) = 34.941; P < .001; Eta² = .040 |  |  |
| T1 | 4.01 (2.13) | 4.55 (1.88) | 3.72 (2.00) | F(1,837) = 11.355; P < .001; Eta² = .026 |  |  |
| **Habit 2** |  |  |  |  |  |  |
| T0 | 4.08 (2.18) | 3.68 (2.35) | 2.92 (2.27) | F(1,837) = 26.378; P < .001; Eta² = .031 |  |  |
| T1 | 3.99 (2.11) | 4.42 (1.92) | 3.58 (2.14) | F(1,837) = 10.647; P < .001; Eta² = .025 |  |  |

^a^WLCG=Waitlist Control Group.

**Appendix Table S2.** *Effect of the Web- and Print interventions on loneliness, perceived age and symptoms of depression in comparison to the WLCG: means, standard deviations and statistics*^a-e^

|  | Experimental group | | | Statistic |  | |
| --- | --- | --- | --- | --- | --- | --- |
|  | WLCG | Web | Print | Time (row for T0) and time*experimental group (row for T1) | |  |
|  |  |  |  |  | |  |
| **Loneliness** |  |  |  |  | |  |
| T0 | 0.28 (.062) | 0.38 (0.68) | 0.45 (0.71) | F(1,837) = .015; P = .904; Eta² < .001 | |  |
| T1 | 0.35 (0.65) | 0.39 (0.64) | 0.38 (0.67) | F(1,837) = 1.884; P = .153; Eta² = .005 | |  |
| **Perceived age** |  |  |  |  | |  |
| T0 | 62.93 (7.56) | 61.93 (7.49) | 60.59 (6.86) | F(1,837) = 11.422; P = .001; Eta² = .016 | |  |
| T1 | 63.59 (6.22) | 62.39 (6.56) | 61.57 (6.16) | F(1,837) = 0.549; P = .578; Eta² = .002 | |  |
| **Symptoms of depression** (**CES-D)** |  |  |  |  | |  |
| T0 | 0.56 (0.35) | 0.55 (0.37) | 0.53 (0.36) | F(1,837) = 14.016; P < .001; Eta² = .019 | |  |
| T1 | 0.58 (0.37) | 0.51 (0.34) | 0.43 (0.31) | F(1,837) = 8.763; P < .001; Eta² = .024 | |  |
