# Supplementary material for: Mitigating Feelings of Loneliness and Depression by Means of Web-Based or Print-Based Physical Activity Interventions: Pooled Analysis of 2 Community-Based Intervention Trials
Source: JMIR Aging. 2022 Aug 9;5(3):e36515. doi: 10.2196/36515 (PMC9399846; doi:10.2196/36515)
Supplement: Multimedia Appendix 2 [file aging_v5i3e36515_app2.docx]

**Appendix Table S1.** *Ordinal least squares regression model results for subjective age (mediator in the loneliness model)*

| Model summary, outcome: subjective age (T1) | | | | | | | |  |
| --- | --- | --- | --- | --- | --- | --- | --- | --- |
| R | R^2^ | MSE | F (HC3) | df_1_ | df_2_ | P |  |  |
| 0.75 | 0.56 | 12.44 | 15.51 | 15.00 | 705.00 | 0.00 |  |  |

Model, outcome: subjective age (T1)

|  | Coefficient | SE(HC3) | t | P | LLCI | ULCI |
| --- | --- | --- | --- | --- | --- | --- |
| Constant | -7.26 | 2.79 | -2.60 | 0.00 | -12.75 | -1.77 |
| Web-based  intervention | 0.29 | 0.71 | 0.42 | 0.68 | -1.10 | 1.69 |
| Print-based intervention | 0.83 | 0.75 | 1.10 | 0.27 | -0.65 | 2.30 |
| Intender stage of change | 1.29 | 0.93 | 1.39 | 0.17 | -0.54 | 3.11 |
| Actor stage of change | 0.64 | 0.77 | 0.83 | 0.41 | -0.87 | 2.16 |
| Interaction web*  intender | -1.10 | 1.01 | -1.08 | 0.28 | -3.09 | 0.90 |
| Interaction web*actor | -1.30 | 0.85 | -1.53 | 0.13 | -2.98 | 0.37 |
| Interaction print*intender | -1.86 | 1.12 | -1.66 | 0.10 | -4.05 | 0.33 |
| Interaction print*actor | -0.77 | 1.03 | -0.75 | 0.46 | -2.78 | 1.25 |
| Loneliness (T0) | 0.09 | 0.22 | 0.42 | 0.68 | -0.34 | 0.53 |
| Subjective age (T0) | 0.58 | 0.06 | 10.21 | 0.00 | 0.47 | 0.69 |
| Sex | 0.32 | 0.29 | 1.08 | 0.28 | -0.26 | 0.89 |
| Age | 0.04 | 0.03 | 1.28 | 0.20 | -0.02 | 0.10 |
| ISCED score | 0.52 | 0.26 | 2.02 | 0.04 | 0.01 | 1.03 |
| Family status | 0.21 | 0.15 | 1.40 | 0.16 | -0.08 | 0.50 |
| BMI score | -0.02 | 0.03 | -0.59 | 0.56 | -0.08 | 0.04 |

*Note*. LLCI: Lower Limit Confidence Interval, ULCI: Upper Limit Confidence Interval.

**Appendix Table S2.** *Ordinal least squares regression model results for loneliness*

| Model summary, outcome: loneliness (T1). | | | | | | | |  |
| --- | --- | --- | --- | --- | --- | --- | --- | --- |
| R | R^2^ | MSE | F (HC3) | df_1_ | df_2_ | P |  |  |
| 0.68 | 0.47 | 0.23 | 46.74 | 10.00 | 710.00 | 0.00 |  |  |

Model, outcome: loneliness (T1).

|  | Coefficient | SE (HC3) | t | P | LLCI | ULCI |
| --- | --- | --- | --- | --- | --- | --- |
| Constant | 0.09 | 0.38 | 0.23 | 0.82 | -0.66 | 0.84 |
| Web-based inter­vention | -0.01 | 0.05 | -0.25 | 0.80 | -0.11 | 0.08 |
| Print-based inter­vention | -0.09 | 0.08 | -1.22 | 0.22 | -0.24 | 0.06 |
| Subjective age (T1) | 0.01 | 0.01 | 2.18 | 0.03 | 0.001 | 0.02 |
| Loneliness (T0) | 0.63 | 0.03 | 18.81 | 0.00 | 0.56 | 0.70 |
| Subjective age (T0) | 0.00 | 0.00 | -0.97 | 0.33 | -0.01 | 0.00 |
| Sex | 0.05 | 0.04 | 1.33 | 0.18 | -0.02 | 0.13 |
| Age | 0.00 | 0.00 | 0.16 | 0.87 | -0.01 | 0.01 |
| ISCED score | 0.00 | 0.04 | -0.26 | 0.79 | -0.08 | 0.06 |
| Family status | 0.03 | 0.02 | 1.21 | 0.22 | -0.02 | 0.07 |
| BMI score | 0.00 | 0.00 | -0.16 | 0.87 | -0.01 | 0.01 |

*Note*. LLCI: Lower limit confidence interval, ulci: upper limit confidence interval.

**Appendix Table S3.** *Ordinal least squares regression model results for subjective age (mediator in the depression model)*

| Model summary, outcome: subjective age (T1) | | | | | | | |  |
| --- | --- | --- | --- | --- | --- | --- | --- | --- |
| R | R^2^ | MSE | F (HC3) | df_1_ | df_2_ | P |  |  |
| 0.75 | 0.56 | 12.44 | 16.12 | 15.00 | 705.00 | 0.00 |  |  |

Model, outcome: subjective age (T1).

|  | Coefficient | SE(HC3) | t | P | LLCI | ULCI |
| --- | --- | --- | --- | --- | --- | --- |
| Constant | -7.24 | 2.82 | -2.57 | 0.01 | -12.78 | -1.71 |
| Web-based intervention | 0.30 | 0.71 | 0.42 | 0.67 | -1.09 | 1.69 |
| Print-based intervention | 0.84 | 0.75 | 1.11 | 0.27 | -0.64 | 2.32 |
| Intender stage of change | 1.28 | 0.93 | 1.38 | 0.17 | -0.54 | 3.09 |
| Actor stage of change | 0.64 | 0.77 | 0.82 | 0.41 | -0.88 | 2.15 |
| Interaction web*intender | -1.08 | 1.01 | -1.07 | 0.29 | -3.07 | 0.90 |
| Interaction web*actor | -1.30 | 0.85 | -1.52 | 0.13 | -2.97 | 0.38 |
| Interaction print* intender | -1.86 | 1.11 | -1.66 | 0.10 | -4.04 | 0.33 |
| Interaction print*actor | -0.76 | 1.03 | -0.74 | 0.46 | -2.78 | 1.25 |
| CES-D (T0) | 0.01 | 0.02 | 0.26 | 0.79 | -0.37 | 0.05 |
| Subjective age (T0) | 0.58 | 0.06 | 10.00 | 0.00 | 0.46 | 0.69 |
| Sex | 0.32 | 0.29 | 1.10 | 0.27 | -0.25 | 0.88 |
| Age | 0.04 | 0.03 | 1.27 | 0.20 | -0.02 | 0.10 |
| ISCED score | 0.52 | 0.26 | 2.02 | 0.04 | 0.02 | 1.03 |
| Family status | 0.22 | 0.14 | 1.51 | 0.13 | -0.07 | 0.50 |
| BMI score | -0.02 | 0.03 | -0.61 | 0.54 | -0.08 | 0.04 |

*Note*. LLCI: Lower limit confidence interval, ulci: upper limit confidence interval.

**Appendix Table S4.** *Ordinal least squares regression model results for depressive symptoms*

| Model summary, outcome: depression (T1). | | | | | | | |  |
| --- | --- | --- | --- | --- | --- | --- | --- | --- |
| R | R^2^ | MSE | F (HC3) | df_1_ | df_2_ | P |  |  |
| 0.81 | 0.66 | 14.30 | 73.82 | 10.00 | 710.00 | 0.00 |  |  |

Model, outcome: depression (T1).

|  | Coefficient | SE (HC3) | t | P | LLCI | ULCI |
| --- | --- | --- | --- | --- | --- | --- |
| Constant | -2.72 | 3.11 | -0.87 | 0.38 | -8.83 | 3.39 |
| Web-based inter­vention | -0.86 | 0.37 | -2.33 | 0.02 | -1.58 | -0.13 |
| Print-based inter­vention | -1.96 | 0.53 | -3.70 | 0.00 | -2.99 | -0.92 |
| Subjective age (T1) | 0.14 | 0.05 | 2.98 | 0.00 | 0.05 | 0.23 |
| CES-D (t0) | 0.70 | 0.03 | 25.30 | 0.00 | 0.64 | 0.75 |
| Subjective age (T0) | -0.07 | 0.03 | -1.87 | 0.06 | -0.13 | 0.00 |
| Sex | 0.37 | 0.31 | 1.22 | 0.22 | -0.23 | 0.97 |
| Age | 0.10 | 0.04 | 2.68 | 0.01 | 0.03 | 0.17 |
| ISCED score | -0.53 | 0.28 | -1.91 | 0.06 | -1.07 | 0.01 |
| Family status | 0.05 | 0.16 | 0.30 | 0.76 | -0.26 | 0.36 |
| BMI score | 0.00 | 0.03 | 0.06 | 0.95 | -0.06 | 0.07 |

*Note*. LLCI: Lower limit confidence interval, ulci: upper limit confidence interval.
